# SOCIAL RESOURCES ARE ASSOCIATED WITH COGNITIVE FUNCTION ACROSS BLACK AND WHITE OLDER ADULTS AND NEIGHBORHOOD TYPES

**DOI:** 10.1093/geroni/igae098.0044

**Published:** 2024-12-31

**Authors:** Ketlyne Sol, Toni Antonucci, Philippa Clarke, Laura Zahodne

**Affiliations:** University of Michigan, Ann Arbor, Michigan, United States; University of Michigan, Ann Arbor, Michigan, United States; University of Michigan, Ann Arbor, Michigan, United States; University of Michigan, Ann Arbor, Michigan, United States

## Abstract

Few studies examine neighborhood resources in healthy cognitive aging despite evidence that living in neighborhoods with lower socioeconomic status (NSES) is associated with increased risk for Alzheimer’s Disease and Related Diseases (ADRD) with persistent race disparities in ADRD and NSES. Fewer studies examine which social resources buffer against the detrimental effects of living in lower-resourced neighborhoods. In this cross-sectional study, multiple linear regressions examined associations between structural and functional social resources and cognition stratified by intersectional groups of participant self-reported race and neighborhood type. Participants were drawn from the Michigan Cognitive Aging Project, a longitudinal epidemiological study of cognitive aging in southeastern Michigan (Mage=64). Global cognition was a composite of five comprehensively-assessed cognitive domains. Participant addresses were linked to year 2017 measures of neighborhood resources from the National Neighborhood Data Archive. Latent class analysis of 2,813 Michigan census tracts identified that most participants lived in tracts characterized as low-resourced (Bn=162=Wn=128) or moderately-resourced (Wn=80>Bn=64). Only among White residents of low-resourced neighborhoods, a larger social network (β=.257, p=.001) was associated with better cognition. Among Black residents of low-resourced neighborhoods, more support in their closest relationship (β=.150, p=.044) was associated with better cognition. Among Black residents of moderately-resourced neighborhoods, more strain from friends (β=-.255, p=.087) was associated with worse cognition. Support from relatives of Black, but not White, residents in both neighborhood types was associated with better cognition. Nuanced, culturally-sensitive interventions may support racially diverse older adults to foster and broaden desired relationships to buffer against adverse cognitive outcomes of neighborhood stressors.

